# Model-to-Data Approach for Deep Learning in Optical Coherence Tomography Intraretinal Fluid Segmentation

**DOI:** 10.1001/jamaophthalmol.2020.2769

**Published:** 2020-08-06

**Authors:** Nihaal Mehta, Cecilia S. Lee, Luísa S. M. Mendonça, Khadija Raza, Phillip X. Braun, Jay S. Duker, Nadia K. Waheed, Aaron Y. Lee

**Affiliations:** 1 New England Eye Center, Tufts Medical Center, Boston, Massachusetts; 2 Warren Alpert Medical School of Brown University, Providence, Rhode Island; 3 Department of Ophthalmology, University of Washington, Seattle; 4 Department of Ophthalmology, Federal University of São Paulo, São Paulo, Brazil; 5 Yale School of Medicine, New Haven, Connecticut

## Abstract

**Importance:**

Amid an explosion of interest in deep learning in medicine, including within ophthalmology, concerns regarding data privacy, security, and sharing are of increasing importance. A model-to-data approach, in which the model itself is transferred rather than data, can circumvent many of these challenges but has not been previously demonstrated in ophthalmology.

**Objective:**

To determine whether a model-to-data deep learning approach (ie, validation of the algorithm without any data transfer) can be applied in ophthalmology.

**Design, Setting, and Participants:**

This single-center cross-sectional study included patients with active exudative age-related macular degeneration undergoing optical coherence tomography (OCT) at the New England Eye Center from August 1, 2018, to February 28, 2019. Data were primarily analyzed from March 1 to June 20, 2019.

**Main Outcomes and Measures:**

Training of the deep learning model, using a model-to-data approach, in recognizing intraretinal fluid (IRF) on OCT B-scans.

**Results:**

The model was trained (learning curve Dice coefficient, >80%) using 400 OCT B-scans from 128 participants (69 female [54%] and 59 male [46%]; mean [SD] age, 77.5 [9.1] years). In comparing the model with manual human grading of IRF pockets, no statistically significant difference in Dice coefficients or intersection over union scores was found (*P* > .05).

**Conclusions and Relevance:**

A model-to-data approach to deep learning applied in ophthalmology avoided many of the traditional hurdles in large-scale deep learning, including data sharing, security, and privacy concerns. Although the clinical relevance of these results is limited at this time, this proof-of-concept study suggests that such a paradigm should be further examined in larger-scale, multicenter deep learning studies.

## Key Points

**Question:**

Can a model-to-data approach be applied to deep learning studies in ophthalmology?

**Findings:**

This cross-sectional study applied a model-to-data approach to deep learning using 400 optical coherence tomography B-scans from patients with active exudative age-related macular degeneration. Without any data transfer between institutions, the model was trained to recognize areas of intraretinal fluid on the scans, and no difference was found in the comparison of the model with manual grading.

**Meaning:**

Although clinical application is at present limited, these results suggest that model-to-data approaches can obviate many of the traditional hurdles in large-scale deep learning projects and may increase application in future ophthalmology studies.

## Teaser

This cross-sectional study assesses whether a model-to-data deep learning approach (ie, validation of the algorithm without any data transfer) can be applied in ophthalmology.

## Introduction

In the last decade, an explosion in the application of machine learning to a wide variety of fields has occurred. *Artificial intelligence*, the umbrella term within which machine learning falls, has been hailed as part of the fourth industrial revolution in human history and a transformative force in clinical medicine.^1,2^Although often used interchangeably, the terms *artificial intelligence*, *machine learning*, *neural networks*, and *deep learning* are not synonymous. Machine learning is a subset of the broader field of artificial intelligence and has been defined as “[giving] computers the ability to learn without being explicitly programmed.”^3^^(p1726)^ Many different machine learning frameworks are available, for example, artificial neural networks, a further subset of which is deep learning. Deep learning specifically uses multiple levels of classification with data features automatically extracted and has proven particularly well-suited for complex data.^4^ Perhaps as a result, deep learning has already been explored, in imaging studies alone, within nearly every field of medicine.^5^

Because of ophthalmology’s dependence on outpatient ancillary testing, machine learning has the potential to be transformative.^3,6,7^ Machine learning and deep learning have already been applied in ophthalmology in a variety of contexts and to a range of clinical conditions, ranging from diabetic retinopathy,^8^ age-related macular degeneration,^9^ and glaucoma^10^ to, more recently, Stargardt disease^11^ and post–small incision lenticule extraction surgical outcomes.^12^ In a major study published in *Nature Medicine*, a deep learning model was successfully trained on almost 15 000 optical coherence tomography (OCT) B-scans to recognize pathologic lesions requiring urgent referral.^13^

**Figure 1.**
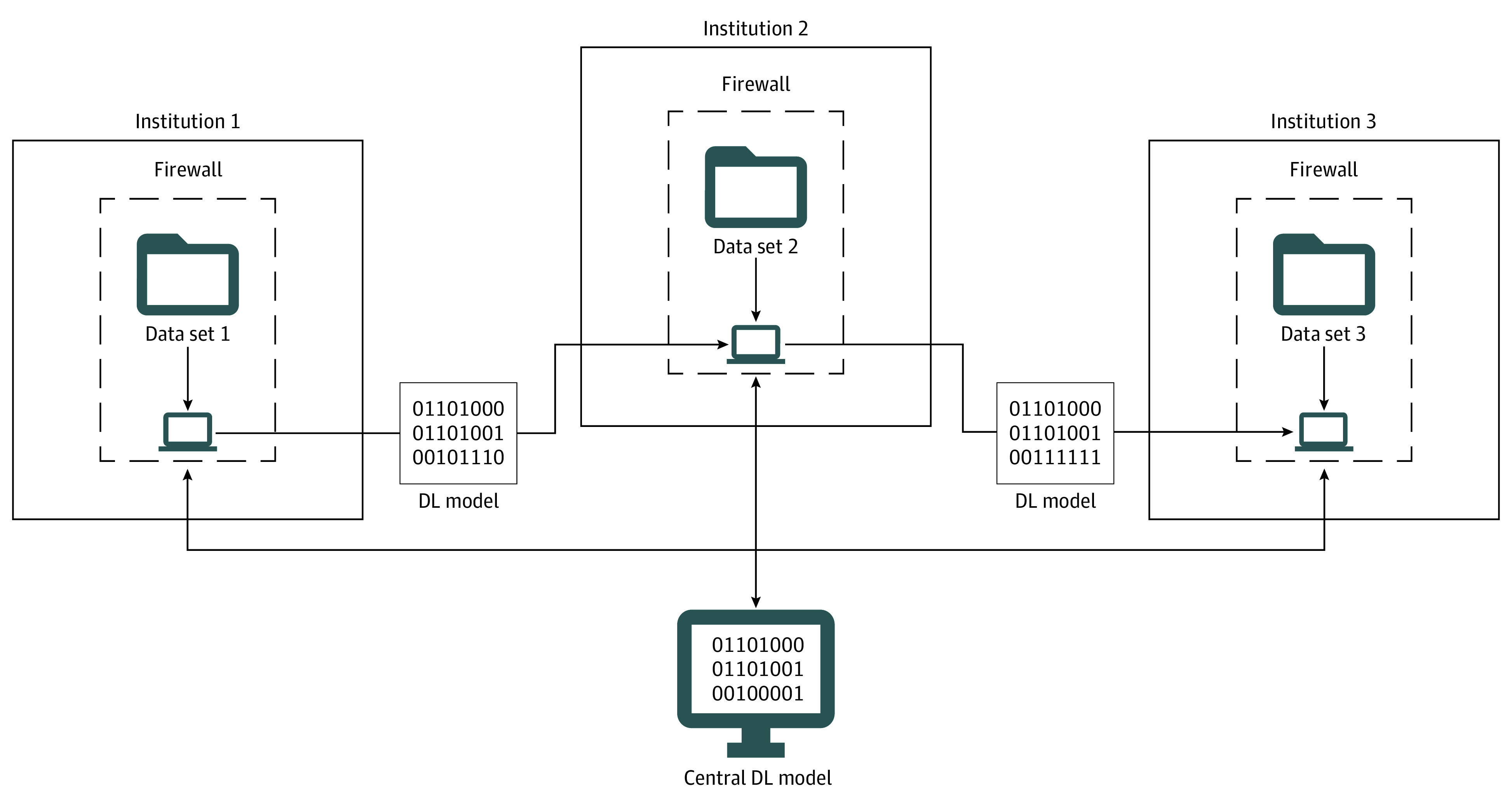
Schematic Description of the Model-to-Data Approach The trained deep learning (DL) model is transferred to a new institution housing its own unique training and test data set, allowing for these data to remain within its firewall. Once trained using the data available at one institution, the updated model alone (without accompanying data) can be transferred to another institution, allowing for rapid iterative training without any data transfer.

However, most deep learning applications require training and test data sets to be applied to a single central model, often with exchange of data between institutions, which may not be possible owing to regulatory processes. On the other hand, data collection sufficient to allow successful deep learning from a single institution is a nontrivial process. In this study, we retrained an existing deep learning network^14^ to segment intraretinal fluid (IRF) on OCT B-scans using a distinct data set at its source center without any data transfer, thus bringing the model to the data (Figure 1).

## Methods

The study protocol was approved by the Tufts Medical Center institutional review board, the source center for the imaging data, and adhered to the tenets of the Declaration of Helsinki^15^ and the Health Insurance Portability and Accountability Act of 1996. The protocol approved the collection of previously obtained and deidentified clinical data without specific consent. This study followed the Strengthening the Reporting of Observational Studies in Epidemiology (STROBE) reporting guideline.

**Figure 2.**
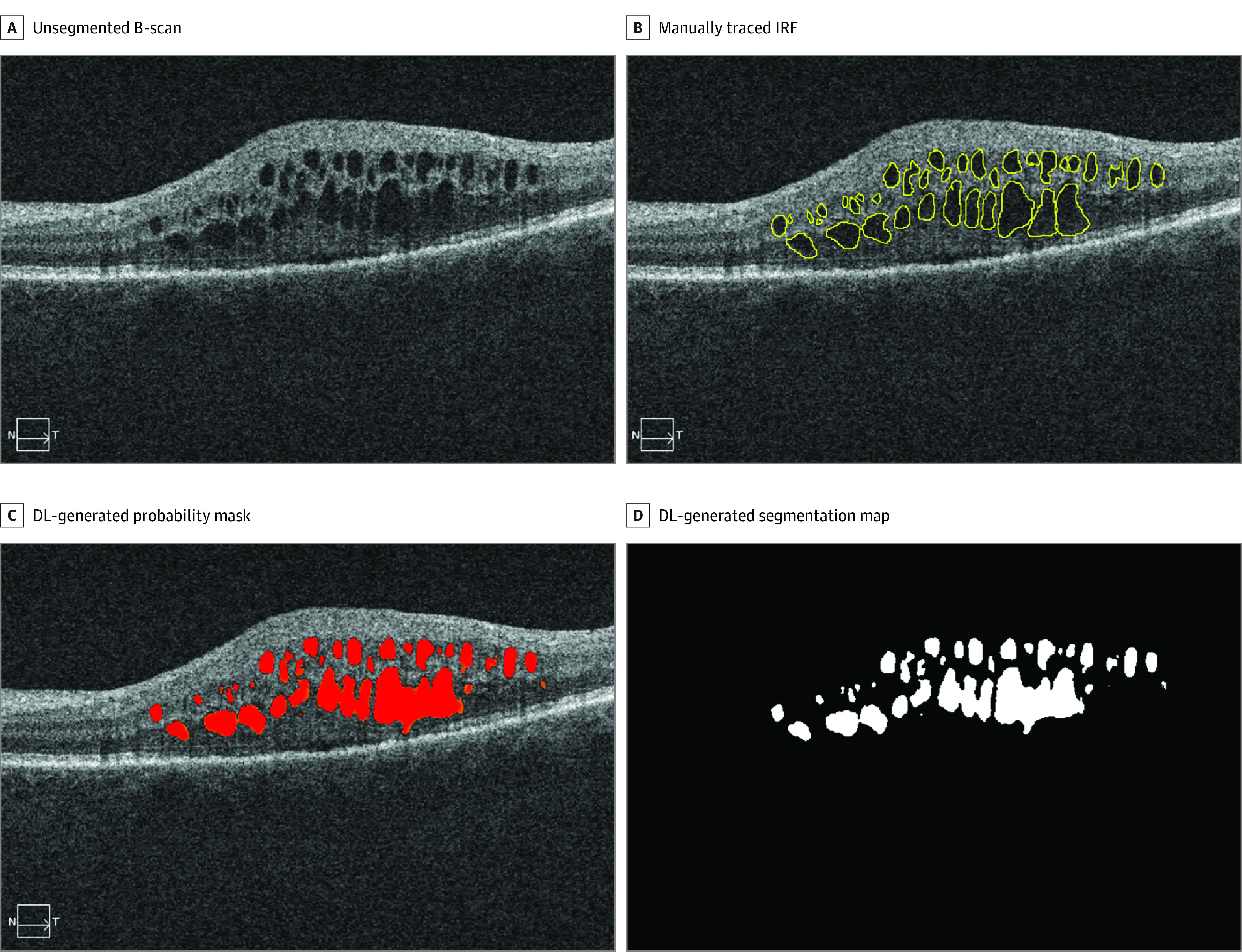
Example Segmentation of Intraretinal Fluid (IRF) The original unsegmented B-scan is shown before (A) and after (B) areas of IRF were manually traced by the human grader. The deep learning (DL)–generated probability mask for areas of IRF (C) and the DL-generated segmentation map (D) are also shown.

We reviewed all OCT volumes of patients with a diagnosis of exudative age-related macular degeneration; images were obtained on the spectral-domain OCT device (Cirrus HD-OCT 5000; Carl Zeiss Meditec) using a macular cube protocol during a 6-month period from August 1, 2018, to February 28, 2019, at the New England Eye Center (NEEC). This scan protocol consisted of 512 A-scans per B-scan and 128 B-scans per volume. The spectral-domain OCT system has an 840-nm central wavelength and the following operational parameters: 68 000 A-scans per second, an A-scan depth of 2.0 mm, an axial resolution of 5 μm, and a transverse resolution of 15 μm. From all eligible OCT volumes in this patient cohort, 400 scans from 58 patients were selected for the training set. An additional 70 scans from 70 patients (ie, 1 scan per patient) that were not included in the training set and collected from a different period than the training set were selected for the test set. Partitioning of the test set was performed at the patient level, and the test data were temporarily segregated from the training set. All scans were reviewed by a trained image reader (N.M.), and individual B-scans displaying IRF were exported as portable network graphics files. Each image was then manually segmented by 3 trained readers (N.M., L.S.M.M., and P.X.B.) who traced areas with IRF,^16^ resulting in a binary segmentation map for each image (Figure 2). All tracing was completed using ImageJ, version 2.0.0 (National Institutes of Health). Of the 400 training images, 70% were randomly designated for training and 30% for validation. Data were segregated at the patient level during the retraining phase.

The primary data analysis occurred from March 1 to June 30, 2019. The model-to-data approach was taken by freezing the model parameters from the prior study^14^ in which a deep learning model was trained to segment IRF on Heidelberg Spectralis OCT B-scans. The model parameters, retraining code, data preprocessing, and code for evaluation were packaged from the University of Washington and transferred using GitHub (https://github.com/uw-biomedical-ml/oct-irf-train). The researchers at the NEEC then downloaded the retraining code with the deep learning model and executed the retraining of the deep learning model. At no point did the researchers at the University of Washington have access to the computer or the OCT images at the NEEC. The model was retrained using OCT images from the spectral-domain OCT device, and segmentations were derived from the NEEC.

Before retraining, the transferred model was first evaluated directly on the test data. During training, OCT images were preprocessed in a manner similar to previously described work.^14^ Briefly, each OCT B-scan in the training set was vertically sliced into narrow (32 × 432 pixels) strip windows. In the training set, overlapping vertical windows were included as a form of offline augmentation. In the validation set, nonoverlapping windows were used for periodically evaluating the performance and determining when overfitting was occurring. The intensities were normalized using the values derived from the prior study. The normalized OCT intensities were then used directly as input into the first convolutional layer of the deep learning model. The output layer of the deep learning model provides a probability of each pixel being IRF.

The deep learning model was retrained using the following hyperparameter settings, similar to the settings used in the previous work^14^: a batch size of 10, learning rate of 1 × 10^−5^, Adam optimizer, and a smoothed Dice coefficient loss. After 30 iterations through the training set, the training was halted and the model was frozen using the highest performance on the validation set. All training was completed on a desktop workstation (Ubuntu, version 18.10; Canonical Ltd) at the NEEC equipped with a commercially available central processing unit (Core i7-5930K 3.50 GhZ; Intel Corp) and a graphics processing unit (GeForce GTX 960; Nvidia Corp).

The model with the lowest validation loss was then frozen, and the final test set of 70 images was evaluated. For each A-scan location, overlapping B-scan windows were used as input into the final model, and for each pixel, the mean of the overlapping inferences was calculated. To create the final binary segmentation masks, a predefined threshold of 0.5 was used to determine whether the mean of the overlapping inference output predicted IRF. The test set was evaluated using whole B-scan level Dice coefficients and intersection over union scores. Differences compared with manual segmentation as the criterion standard were assessed using the Wilcoxon rank sum test and 1-way Kruskal-Wallis test, given the nonnormal distribution of the data corrected for multiple comparisons. All statistics were performed using R, version 3.3.2 (R Project for Statistical Computing).

## Results

The deep learning model was successfully packaged, transported, and retrained using the 400 segmented IRF images from 128 patients (69 female [54%] and 59 male [46%]; mean [SD] age, 77.5 [9.1] years) without any transfer of imaging data between institutions. Figure 1 displays a schematic of the model-to-data framework we used. Figure 2 shows an example image with IRF after manual human tracing and segmentation by the deep learning model.

**Figure 3.**
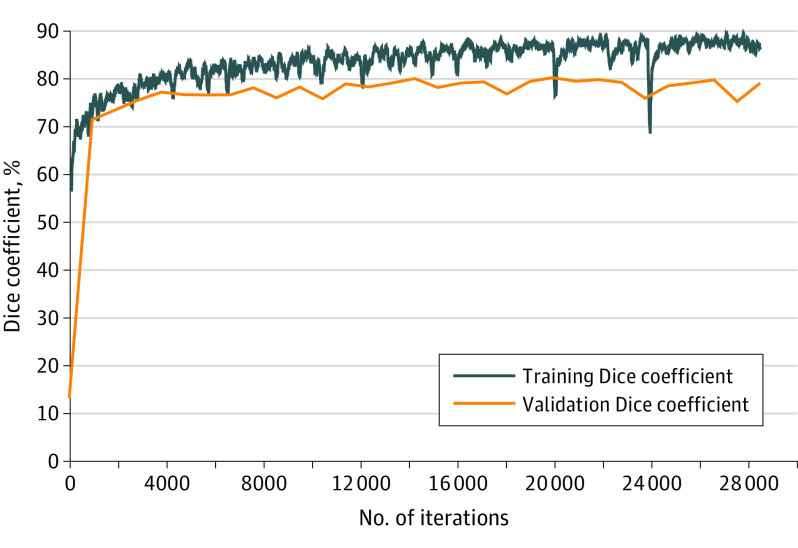
Deep Learning Network Learning Curve Generated During Training of the Model

**Figure 4.**
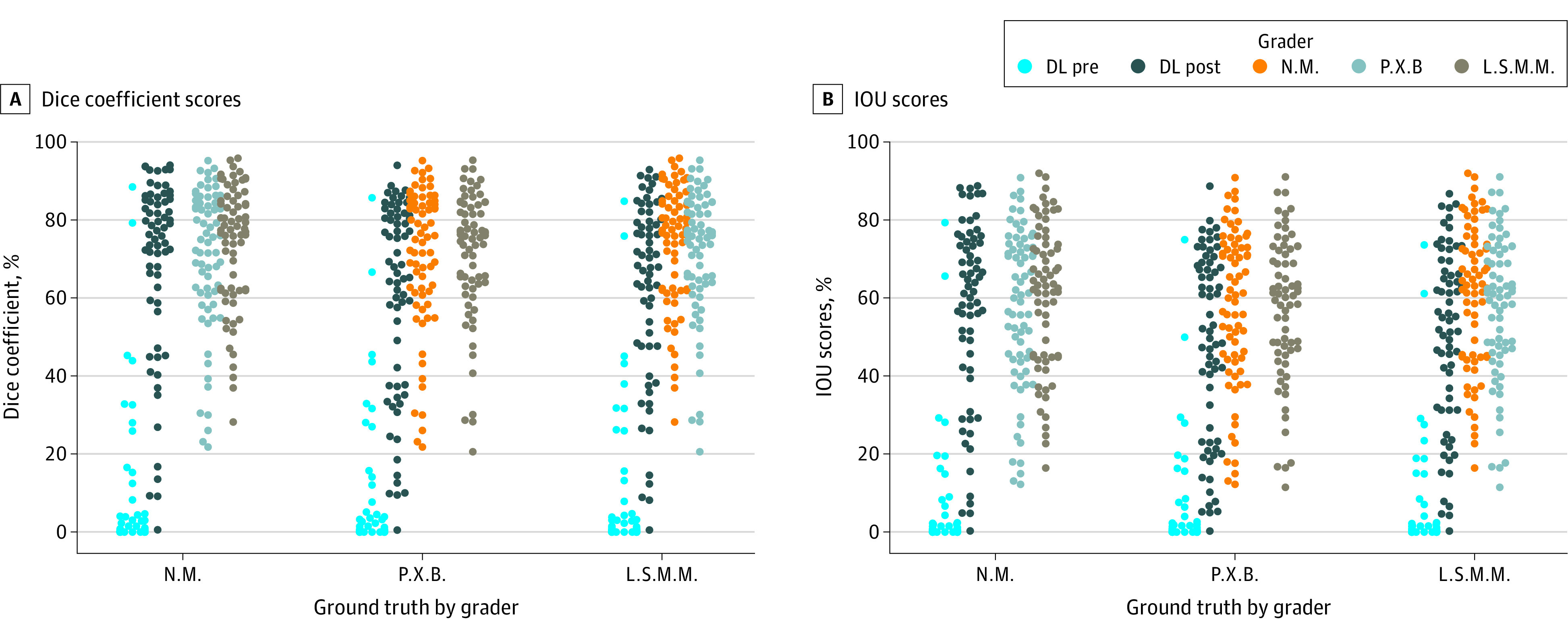
Swarm Plots of the Dice Coefficient and Intersection Over Union Scores The distributions of Dice coefficient scores (A) and intersection over union (IOU) scores (B) for the deep learning model (after transferring but before retraining [DL pre] and after transferring and retraining [DL post]) and each human grader (N.M., P.X.B., and L.S.M.M.) are compared, with each human grader as the criterion standard.

The learning curve of the network during retraining as measured by Dice coefficient is displayed in Figure 3. Dice scores were not statistically different between deep learning and human graders by reference standard rotation (*P* = .09 for L.S.M.M., *P* > .99 for N.M., and *P* = .12 for P.X.B. by Bonferroni-corrected Kruskal-Wallis test). Similarly, intersection over union scores was not statistically different between deep learning and human graders (*P* = .10 for L.S.M.M., *P* > .99 for N.M, and *P* = .12 for P.X.B.). (Figure 4). The performance of the model before retraining is also shown in Figure 4. The differences in Dice coefficient and intersection over union scores for the model before vs after retraining were all statistically significant (*P* < 2.2 × 10^−16^) and showed that the performance of the model after retraining were all statistically significantly better than before retraining, regardless of which human grader was chosen as the criterion standard. All training and evaluation code, model architecture, and model weights are available at GitHub (https://github.com/uw-biomedical-ml/oct-irf-train).

## Discussion

Without any data transfer, we trained, validated, and tested a previously reported deep learning model that quantifies the area of IRF in OCT using a new data set. The deep learning network performance did not differ statistically compared with that of human graders (ie, compared with each single human grader as a criterion standard). The ability of this model to accurately segment IRF in structural OCT images of patients with diabetic macular edema, exudative age-related macular degeneration, and retinal vein occlusions has been previously demonstrated, with the model achieving a maximal cross-validation Dice coefficient of 0.911 compared with manual segmentation.^14^ Such an approach could be extended to quantify fluid volumes in 3-dimensional cube scans, allowing for tracking of fluid volume over time and response to treatment in various retinal diseases.^16^ It is important to note that this forms only 1 of the building blocks that will allow approaches such as these to be used clinically. A recent review of deep learning studies of medical imaging^17^ highlighted several shortcomings in the existing literature, chiefly a lack of randomized clinical trials, which need to be addressed before approaches such as these can be used in the clinical setting. As an example, using our approach, robust clinical studies correlating fluid volume to treatment efficacy or to visual outcomes would be needed before this algorithm could be of clinical value.

In the present study, our approach represents, to our knowledge, the first application in the field of ophthalmology of a packaged, transportable deep learning model to the data between 2 institutions (Figure 1). In the past several years, amid an explosion of artificial intelligence, similar interest has grown in decentralized approaches to deep learning training.^18^ Libraries such as UNet and TensorFlow (https://www.tensorflow.org/) have greatly accelerated the development and accessibility of deep learning. More recently, the Open Neural Network Exchange format (https://onnx.ai/), a collaborative effort between Facebook and Microsoft Corporation, created an open-source platform to facilitate interoperability and exchange of artificial intelligence models, and Google’s announcement of their Deep Learning Containers (https://cloud.google.com/ai-platform/deep-learning-containers/) is an effort toward the same goal.

Despite the incredible promise and successes in deep learning approaches, a major challenge in medical applications of deep learning remains the large amount of accurate data needed for successful training—an “insatiable appetite for training data.”^19^^(p118)^ Deep learning studies specific to medical imaging have shown improved model performance with increasingly large data sets and unacceptably poorer performance with small data sets.^20,21^ The precise amount of data required is largely contingent on the complexity of the deep learning model and the structure of the data; however, it is clear that deep learning approaches are unlikely to be successful without large data sets, especially in medical applications in which a high degree of accuracy is required. Data scarcity thus represents one of the chief rate-limiting steps for applying deep learning within medical and health care contexts despite the incredible promise.^22,23,24,25^

In general, obtaining adequately sized data sets requires compiling data from multiple clinical sites and transferring to a single site where the computational model is housed. Although this is technically feasible, compiling data from multiple sources into a single large data set has several challenges, including the time and resources needed to transfer data. The computational resources and time needed to transfer the large data needed for deep learning applications, such as imaging or video data, can be considerable, in addition to the often massive computational needs for deep learning training itself. Of note, the largest-scale deep learning study in ophthalmology thus far used data from 32 clinical sites within the Moorfields Eye Hospital NHS (National Health Service) Foundation Trust.^13^ This study also used Google DeepMind, a historically powerful system.^26^ Achieving data sets of this scale (particularly without the benefits of a nationalized health system, as is the case in the United States) will likely pose major challenges, and computational power on the scale of DeepMind is out of the reach of all but the largest organizations and institutions. Minimizing the resources needed for deep learning studies could thus significantly increase its accessibility. Additional hurdles include security and privacy risks in transferring potentially identifiable data, the potential need for multiple data sharing agreements, and obtaining appropriate permissions; both the International Committee of Medical Journal Editors^27^ and the Institute of Medicine^28^ have recently emphasized the importance of ethical data sharing. The European Union’s General Data Protection Regulation^29^ and the United States Health Insurance Portability and Accountability Act^30^ are examples of the international legal hurdles to sharing of clinical data. Various methods of improving the efficiency of data sharing and of augmenting existing data training features have been proposed and implemented.^31^ These approaches, however, still rely on an essentially data-to-model paradigm. Other techniques include federated learning, in which training data remain distributed and updates to a centralized model are shared, and differential privacy, whereby a model is indirectly trained on multiple data sets that are disjointed from the underlying sensitive data.^32,33^

A more advantageous solution would be a model-to-data approach, as applied in the present study (Figure 1). The model-to-data paradigm, in which “data remain stationary with models moving to the data,” has been previously described by Guinney and Saez-Rodriguez^34^^(p392)^ and has been successfully applied in a number of large-scale data challenges, including the Digital Mammography DREAM Challenge. The model-to-data framework can obviate many of the hurdles intrinsic to the traditional data sharing approach and thereby not only improve the efficiency with which deep learning projects could be completed but also potentially allow data that were not initially collected with permission for sharing to be used. This in turn would increase data availability, improve the diversity of data sets, and augment the abilities of resulting deep learning models. Particularly in a clinical context, a model-to-data approach mitigates many of the major challenges for clinical use of deep learning, including data transfer. We trained a deep learning model on a central system at one institution and then retrained it on a distinct computer system at a separate institution; no clinical data were transferred between institutions, and the study was completed without researchers exposed to the other institution’s clinical data. In the future, model-to-data could allow for central construction of a robust deep learning model, followed by distribution to multiple clinical sites where distributed training can take place.

A similar distributed deep learning approach was described and simulated, using a single data set and without actual model distribution to a distinct institution, by Chang et al.^35^ In that study, a model was trained to classify images—including color fundus photographs of patients with diabetic retinopathy—using model distribution among 4 simulated institutions. Our study extends the approach proposed by Guinney and Saez-Rodriguez^34^ and simulated by Chang et al^35^ by demonstrating the feasibility of a model-to-data approach by retraining deep learning networks across 2 distinct institutions, with training data from separate patient populations and different machines as the input source. The present study is thus primarily a proof of concept for the model-to-data approach in ophthalmology: that of packaging an easily shared deep learning model and bringing it to the data. Our findings show that collaborative machine learning can be completed in ophthalmology without any data leaving the home institution. If extended, this strategy could allow for open-source, collaborative training networks among multiple institutions, each of which would generate models with distinct training weights. These weights could then be compiled to create a single, multicenter, validated deep learning model.

Emerging ethical issues are related to data privacy for deep learning applications. Our approach satisfies and has distinct advantages under the conventional standard for data sharing and patient confidentiality, namely that data, even if unidentifiable, should not be shared outside an institution without specific permissions.^36^ Bringing the model to the data is—from a privacy, confidentiality, and security perspective—much easier in this regard. However, as deep learning models become more accurately reflective of the data from which they were trained, the question has arisen of whether training data could be reconstructed from the trained deep learning model. This possibility, termed *model inversion*, was first introduced in the context of medical applications for deep learning and is part of the broader set of emerging privacy and security concerns.^37,38^ Model inversion has recently been explored further, with several studies suggesting that deep learning models are vulnerable to such “inversion attacks” unless specifically designed to mitigate their susceptibilities.^39,40^ However, the extent of the risk posed by model inversion in allowing for reconstruction of training data is not entirely clear; other information about the original data may be needed to reconstruct it to any meaningful degree. Further studies are needed to better understand model inversion threats. For example, the threat posed by using model inversion to partially reconstruct highly identifiable information, such as a photograph of a face,^40^ may be less consequential for imaging data such as an OCT B-scan. However, to the extent that deep learning models are susceptible, our model-to-data approach does not obviate vulnerability to inversion. Unless other approaches to security and privacy, such as differential privacy, are incorporated, a deep learning model produced by a model-to-data approach could still theoretically be inverted. Consequently, a multifaceted approach will likely be necessary in the construction of adequately secure future deep learning models.

### Limitations

There were several limitations to our study. Training of the existing model was completed at a single site using data from 1 device. To explore the model’s potential, this approach needs to be extended in multicenter and multidevice studies beyond this initial proof-of-concept study. Moreover, although our deep learning model showed no statistically significant differences in performance vs human grading, its performance may have been improved with an even larger data set. Future studies should further explore the potential of a model-to-data approach, including with larger data sets, different types of imaging data, and multicenter data. Studies completed in a more clinical setting will be valuable in assessing whether this approach has application in clinical practice.

## Conclusions

A model-to-data approach to deep learning was demonstrated for the first time, to our knowledge, in ophthalmology. Using this approach, the performance of the deep learning model was trained and showed no statistically significant difference in quantifying the intraretinal fluid pockets in OCT compared with human manual grading. Such a paradigm has the potential to more easily facilitate large-scale and multicenter deep learning studies.
